# A New Diagnosis of a Genetic Disorder in a Patient Presenting with Bilateral Upper Extremity Neuropathy

**DOI:** 10.5811/cpcem.2017.11.35867

**Published:** 2018-01-19

**Authors:** Todd M. Ruttenberg, Alexander D. Miller

**Affiliations:** Naval Medical Center San Diego, Department of Emergency Medicine, San Diego, California

## Abstract

A 20-year-old male United States Marine Corps recruit was admitted to the emergency department with a two-week history of profound, bilateral upper-extremity weakness and numbness. Initially thought to be the result of his military training, the cause was ultimately determined to be genetic. This case represents a rare cause of a somewhat common presenting symptom: chronic symmetric polyneuropathy.

## INTRODUCTION

Hereditary neuropathy with a liability to pressure palsies (HNPP) is a well-established, albeit rare, diagnosis with recurrent episodes of nerve demyelination. Affected patients typically present with isolated nerve palsies in areas frequently affected by compression or trauma, even of a trivial nature. HNPP is an autosomal dominant disorder most often associated with peripheral myelin protein 22 (PMP22) gene mutations, and may present de novo as well.^1^,^2^

Although not a diagnosis requiring emergent intervention, HNPP may present with a dramatic and confusing constellation of symptoms and should be considered after other life-threatening processes are ruled out. HNPP carries with it a significant impact on the quality of the life for those whom it affects, therefore making its existence on our collective differential diagnosis of the utmost importance. The combination of peripheral neuropathy and upper extremity weakness carries a broad differential diagnosis, many of which have immediate treatment needs. Some may require hospitalization and further diagnostic evaluation to ensure a minimization of possible life threats and permanent neurologic deficits.

## CASE REPORT

A 20-year-old male United States Marine Corps (USMC) recruit presented to the emergency department (ED) with a two-week history of stable yet profound bilateral, painless, upper extremity weakness, left greater than right, and a patchy, non-dermatomal sensory deficit, to the upper extremities. Three weeks prior to presentation, the USMC recruit in training at the Marine Corps Recruit Depot first noticed a continuous numbness on the dorsal aspect of his left hand between the thumb and index finger. He thought little of the numbness as it did not impede him.

The following week, he noticed a sudden-onset, profound, right-hand grip strength weakness while in the prone position during rifle training as a right-handed shooter. He was seen in clinic, diagnosed with “backpack palsy” – a compressive neuropathy of the brachial plexus – and returned to rifle training. He then completed another day of rifle training in the prone position, this time as a left-handed shooter. Soon after, he developed weakness to the left upper extremity when in abduction and flexion. His training was halted to allow for time to assess if the presumed “backpack palsy” would improve. Ten days passed with no improvement, and he noticed that the weakness worsened with continued use. At this point the patient was having difficulties with activities of daily living, such as pulling up and fastening his pants. He was then sent to the ED for further evaluation and management.

Upon arrival to the ED, the patient was in no apparent distress and was pain free. He denied headache, neck pain, changes in vision, difficulty breathing, lower extremity weakness, sensory loss, or loss of bowel/bladder control. Vital signs were blood pressure 108/62 millimeters mercury, heart rate 56 beats per minute, respiratory rate 16 breaths per minute, oxygen saturation of 99% on room air, and temperature 97.7ºFahrenheit. His physical exam revealed a behaviorally appropriate patient with normal cranial nerves, normal gait, normal lower extremity strength and sensation and patellar tendon reflexes, negative Romberg test, and painless extremities with less than two-second capillary refill globally. Physical exam pertinent positive findings included asymmetric upper extremity strength testing and sensory deficits to light touch with decreased triceps deep-tendon reflexes bilaterally for the upper extremities.

Given this patient’s non-dermatomal presentation, atraumatic history, lack of associated symptoms, and relatively stable time course the need for hospitalization was considered less likely. Neurology was consulted and evaluated the patient at bedside, recommending an outpatient electromyogram (EMG) in clinic in one to three days for presumed multifocal lower motor neuron process. Prior to follow-up the patient developed sharp shooting pains localized to the left elbow without any known trigger, and he was directly admitted to the hospital from clinic to expedite testing.

Laboratories for autoimmune, musculoskeletal, infectious, and endocrine processes were unremarkable. Magnetic resonance imaging (MRI) of the cervical spine was essentially normal and did not demonstrate demyelinating lesions. EMG demonstrated bilateral compressive median neuropathy at the wrist, bilateral ulnar neuropathy at the elbow, and left upper brachial plexopathy with significant axonal features consistent with HNPP. Confirmatory genetic testing of the PMP22 gene via a diagnostics service was sent for verification and confirmed the diagnosis.

## DISCUSSION

Patients presenting with HNPP and hereditary neuropathies present an intriguing challenge for the emergency physician. Patients with these conditions comprise only a small segment of the large number of patients presenting with peripheral neuropathy. In fact, chronic symmetric symptomatic polyneuropathy in patients is somewhat common and may occur in up to 8% of the general population. By contrast, hereditary motor sensory neuropathies like Charcot-Marie-Tooth disease and HNPP, which affect the peripheral nerves and anterior horn cells of the spinal cord, only have a variable prevalence from 0.008 – 0.11%.^3^ Thus, it affects an estimated 150,000 people in the U.S.^4^ HNPP itself is well documented in the literature, and has an estimated prevalence of at least 0.016% of the general population. Due to the insidious nature of HNPP, and the fact that many patients will present with subclinical symptoms, a mild or late onset, or varying levels of severity from an early age, the true prevalence is most likely underestimated.^5^

Peripheral neuropathy occurs in several common and rare disease states with diverse pathology and varying degrees of severity to the individual patient. This poses a significant challenge to physicians who are attempting to identify and treat these conditions. In our patient, we first considered all the potential emergent diagnoses. This ultimately led to the increased likelihood of first a multifocal lower motor neuron process and finally to the ultimate diagnosis of HNPP through neurology follow-up, EMG studies and genetic testing.

CPC-EM Capsule

What do we already know about this clinical entity?

Hereditary neuropathy with a liability to pressure palsies (HNPP) is documented in the neurology literature. Patients present with nerve palsies in areas affected by compression or trauma, even of a trivial nature.

What makes this presentation of disease reportable?

Although a rare entity, this case provides a good, basic review of peripheral neuropathies.

What is the major learning point?

This case highlights the difficulty in identifying HNPP and provides a review of the patient with non-dermatomal peripheral neurologic complaints.

How might this improve emergency medicine practice?

Provides the practitioner with the knowledge base to identify HNPP and provide the patient with informed counsel.

In this case, central hemorrhagic and ischemic processes were initially considered and then excluded as the patient presented with a history and physical exam inconsistent with the dermatomal and progressive presentation consistent with these diagnoses. As well, a central venous thrombosis was also initially considered as it may present with a non-dermatomal deficit presentation; again, this was deemed highly unlikely secondary to the lack of historical relevance, lack of cranial nerve involvement or seizure activity, and lack of concurrent headache symptoms.

Syringomyelia was also carefully considered but as the patient presented without corresponding deficits to any particular nerve trunk or nerve root level this diagnosis was also unlikely. Guillain-Barré syndrome was also considered but was not consistent with history or physical exam findings. Toxic exposure and neuropathy was considered a possibility, but lack of exposure history made this implausible. Finally, the hypo-reflexia, down-going plantar reflex, and lack of a defined sensory level defect, lower extremity involvement, or traumatic history, made a compressive cervical spine lesion also unlikely. This ultimately led to the consideration of a hereditary neuropathy as the most likely lower motor neuron process responsible for our patient presentation.

According to the National Institute of Neurological Disorders and Stroke, the classification of hereditary neuropathies is divided into four major subcategories: hereditary motor and sensory neuropathy; hereditary sensory neuropathy; hereditary motor neuropathy; and hereditary sensory and autonomic neuropathy. The most common type is Charcot-Marie-Tooth disease, one of the hereditary motor and sensory neuropathies. Symptoms of the hereditary neuropathies vary according to the type and may include sensory symptoms such as numbness, tingling and pain in the feet and hands; or motor symptoms such as weakness and loss of muscle bulk, particularly in the lower leg and feet muscles. Certain types of hereditary neuropathies can affect the autonomic nerves, resulting in impaired sweating, postural hypotension, or insensitivity to pain.

Some people may have foot deformities such as high arches and hammertoes, thin calf muscles (having the appearance of an inverted champagne bottle) or scoliosis (curvature of the spine). The symptoms of hereditary neuropathies may be apparent at birth or appear in middle or late life. They can vary among different family members, with some family members being more severely affected than others. Hereditary neuropathies can be diagnosed by blood tests for genetic testing, nerve conduction studies, and by nerve biopsies.^6^,^7^ Once the diagnosis is made, it is important to know that hereditary neuropathies like HNPP have no standard treatments. Treatment is mainly symptomatic and supportive. Medical treatment includes physical therapy and, if needed, pain medication.^5^,^6^,^7^

## CONCLUSION

This case demonstrates a challenge to the emergency physician as the relative rarity of hereditary neuropathy inhibits prompt recognition and affords only the most astute practitioners confidence in its likelihood. Lack of familiarity with hereditary neuropathies may lead to the performance of unnecessary testing, thus placing patients at undue risk and inhibiting our ability to provide competent and informed recommendations to patients facing an uncertain prognosis.
